# Mortality from Selected Cancers among Brazilian Mechanics

**DOI:** 10.31557/APJCP.2020.21.6.1779

**Published:** 2020-06

**Authors:** Aline Souza Espindola Santos, Amanda Alzira Friaes Martins, Eline Simões Gonçalves, Armando Meyer

**Affiliations:** 1 *Occupational and Environmental Health Branch, Public Health Institute, Federal University of Rio de Janeiro, Rio de Janeiro, Brazil. *; 2 *Center for Studies on Workers’ Health and Human Ecology, National School of Public Health, Oswaldo Cruz Foundation, Rio de Janeiro, Brazil. *

**Keywords:** Cancer, occupational epidemiology, mechanics, mortality

## Abstract

**Introduction:**

Mechanics are exposed to known human carcinogens. This study aimed to compare mortality from selected cancers between male mechanics and the general population of the South and Southeast regions of Brazil.

**Methods:**

Data on deaths, occurred between 2006-2017, among male mechanics and the general population, were obtained from the Mortality Information System. Occupations were classified using the Brazilian Classification of Occupations. Mortality Odds Ratio (MOR) and confidence intervals (95%) for selected cancers among mechanics, stratified by age (30-49, 50-69 years), race, and education compared to the general population, were estimated using logistic regression models.

**Results:**

In general, mechanics showed higher mortality from oropharynx, hypopharynx, larynx, lung and bladder cancers, but lower mortality for all leukemias. Oropharynx and larynx cancer mortality risk was slightly higher among older mechanics, while hypopharynx cancer mortality was more noticeable among the youngest. Lower mortality from all leukemias was observed only among younger mechanics. Mortality by oropharynx and larynx cancers were higher among white mechanics. They were also the only ones to experience higher mortality by hypopharynx cancer, while lung cancer mortality were increased only among non-white ones. Mechanics of all educational levels were more likely to die by the oropharynx cancer. Those with 1-7 and 8 or more years of schooling also showed excess of death by the cancers of larynx and all leukemias. Significantly higher mortality by pancreas cancer was only observed among mechanics with no education, while those with 1-7 years of schooling showed higher risk to die by lung and bladder cancers. Those with 8 or more years of schooling show increased mortality risk for hypopharynx cancer. Increased mortality risk for myeloid leukemia was only observed when stratified by region of residence.

**Conclusion:**

Results of our study suggest a positive association between mechanic occupation and some specific cancers.

## Introduction

Mechanic is a professional potentially exposed to several chemical compounds (Ataro et al., 2019). Some of these chemicals or mixture of chemicals such as diesel engine exhaust, polycyclic aromatic hydrocarbons, asbestos, trichloroethylene and benzene are classified as carcinogenic to humans (Group 1) by the International Agency for Research on Cancer (IARC, 2019). Others, like lead, styrene and tetrachloroethylene are probable human carcinogens (Group 2A), while gasoline, carbon tetrachloride, and metallic fumes containing nickel and chromium are possible human carcinogens (Group 2B) (IARC, 2019). 

Exposure to solvents, metallic fumes, diesel exhaust particles and gases and dust both by inhalation and skin contact occur during degreasing, cleaning, cutting, disassembling/assembling, repair and welding of mechanic parts (Udonwa et al., 2009; ILO, 2012). Oral exposure is often associated with eating, drinking, and smoking on work site without previous hand washing or additionally when particles expelled by the mucociliary transport are swallowed (Christopher et al., 2007).

Several cancers have been linked to the chemicals and tasks involved in the day-by-day mechanic’s activities. For instance, occupational diesel exhaust, polycyclic aromatic hydrocarbons or asbestos exposure is associated with increased lung and bladder cancer risk (Rota et al., 2014; Olsson et al., 2011; Nielsen et al., 2014). Exposure to chlorinated solvent and benzene in different occupation activities are suspected to increase the risk of larynx, hypopharynx, oropharynx cancer, and leukaemia, specially the acute myeloid type (Radican et al., 2008; Barul et al., 2017; Khalade et al., 2010; Santos et al., 2016). Studies have also suggested that metalworking occupations show a higher risk for bladder, and nasopharyngeal cancer (Marsh et al., 2007; Khlifi et al., 2013). 

Occupational cancer represents the main cause of work-related death worldwide and the estimates suggest an increase in its incidence (Lavicoli et al., 2019). The use of new chemicals and technologies without proper information on their carcinogenicity may be associated with the increasing cancer risk in specific occupational groups, which requires a constant attention from the scientific community. Despite all these evidence of carcinogenic risk factors involved in mechanic occupation, few and old studies have investigated the association between this occupation and cancer, especially in developing countries like Brazil (Silva et al., 2000; Andreotti et al., 2006). In this study, we compared the chance of death by specific cancers between mechanics and the general population in the South and Southeast regions of Brazil from 1996 to 2017.

## Materials and Methods

*Methods*

In this study, we compared the cancer mortality data on mechanics with that from the general population for the period of 2006 to 2017. Mortality data on cancer and all other causes of death for mechanics and the general population were obtained from the Brazilian Mortality Information System of the Brazilian Health Informatics Department. Our analyses were restricted to the Brazilian South and Southeast regions, which are the ones with better mortality data quality (Lima and Queiroz, 2014). In these regions, from 2000 to 2010, the coverage and completeness of records were close to 100%, whereas the average percentage of ill-defined causes of death were the smallest (5.3 and 9.5%, respectively) when compared to the other Brazilian regions. In addition, South and Southeast are the most developed Brazilian regions, and the quality of cancer diagnosis information is superior due to better access to healthcare and diagnosis tests.

Individuals whose occupation was coded as retired, pensioners, undeclared occupation, and unemployed were excluded. Occupational data were retrieved according to Brazilian Classification of Occupations (CBO version 2002) using all the codes for mechanic occupations. We examined the excess mortality among male mechanics aged 30-69 years compared to the general population stratified by age, race, education, and region. 

Distribution of sociodemographic characteristics of deaths between mechanics and the Brazilian general population by age groups (30-49 and 50-69 years), education (none, 1-7 years, 8 or more years of schooling), race (white, non-white) and region of residence (South and Southeast) were compared using Pearson’s chi-square test. Frequency of selected cancers (hypopharynx: ICD-10=C10; nasopharynx: ICD-10=C13; larynx: ICD-10=C11; lung: ICD-10=C32; bladder: ICD=C67; myeloid leukemia (ML) ICD-10=C92; and all leukemias: ICD-10=C91-95) between mechanics and the general population were described. Mortality odds ratios (MOR) stratified by age, education, and race were calculated as a measure of association according to the methodology proposed by Miettinen and Wang (1981). MOR is based on the ratio of mortality odds between the selected disease and selected causes of death for a specific group (mechanics) compared to a reference group (general population). Advantages and disadvantages of calculating MOR instead of other measures of association have been previously discussed elsewhere (Meyer et al., 2003).

Mortality data used in the current study are non-personally identifiable and publicly available through the Brazilian Public Health System’s Informatics Department (http://datasus.saude.gov.br/) and, therefore, did not require IRB review.

## Results

Table 1 shows the mortality for selected cancers stratified by sociodemographic characteristics among mechanics and the general population of Brazilian South and Southeast regions. Mechanics and the general population significantly differed in terms of education (p<0.001). Mechanics with 1-7 years of schooling were more likely to die by the studied cancers. On the other hand, among the illiterates or those with 8 years of schooling or more, mortality was higher in the general population. Mortality of mechanics and the general population was also significantly different regarding region of residence (p<0.001). Mechanics’ mortality was higher in the Southeast, but not in the South. Mechanics and the general population did not show differences in the distribution of age (p=0.83) and ethnicity (p=0.49).

Mortality odds ratios for selected cancers among mechanics compared with the general population are shown in Table 2. Mechanics were at higher risk to die by the cancers of oropharynx (MOR:1.84, 95%CI: 1.66-2.11), hypopharynx (MOR:1.39, 95%CI:1.07-1.81), larynx (MOR:1.45, 95%CI:1.32-1.59), lung (MOR:1.07, 95%CI:1.01-1.12), and bladder (MOR:1.16, 95% CI:1.02-1.32). By contrast, leukemia mortality was lower among mechanics (MOR:0.85, 95%CI:0.75-0.96) than in the general population.

Table 3 presents the stratification of selected cancer mortality risk among mechanics and the general population by age and region of residence. Data combined for both regions of residence, but stratified by age, showed an increased risk of oropharynx cancer among younger (30-49 years old) mechanics (MOR:1.66, 95% CI:1.24-2.22) and an even slightly higher risk of this cancer among older mechanics (50-69 years old; MOR:1.85, 95%CI:1.60-2.13), if compared to the general population. Younger and older mechanics were also at higher risk to die by hypopharynx cancer, with a slightly higher mortality odds ratio observed for the former (MOR:1.90, 95%CI:1.13-3.29 and MOR:1.40, 95%CI:1.03-1.90, respectively). Larynx cancer mortality risk was also higher among mechanics (30-49 years old; MOR:1.31, 95%CI:1.01-1.70 and 50-69 years old; MOR:1.42, 95%CI:1.27-1.59) than in the general population. Finally, in the opposite direction, we observed a significantly lower risk of death by all leukemias among 30-49 years old mechanics (MOR: 0.67, 95%CI: 0.51-0.90). Further stratification by region of residence revealed that cancer mortality risk among Brazilian mechanics from South and Southeast presents some similarities, but also few differences, when compared to the general population. Mechanics in the South and Southeast regions were at higher risk to die by oropharynx cancer, but their risks differ according to age. While in the South, this risk was a bit higher among older mechanics (30-49 years old; MOR:1.58, 95%CI:1.11-2.23 and 50-69 years old; MOR:1.91, 95%CI:1.67-2.19), in the Southeast the magnitude was higher among the youngers (30-49 years old; MOR:1.88, 95%CI:1.10-3.21 and 50-69 years old; MOR:1.60, 95%CI:1.17-2.19). Hypopharynx cancer mortality was significantly increased among 30-49 years old mechanics in the South region (MOR: 2.14, 95%CI: 1.13-4.03), but this significant increase wasn’t seen in the Southeast. Another increasing mortality risk observed only in the South region was for myeloid leukemia among 50-69 years old mechanics (MOR: 1.36; 95%CI: 1.04-1.79). On the other hand, leukemia mortality risk was significantly lower among younger mechanics in the same region (MOR: 0.68; 95%CI: 0.49-0.95),but not among those living in the Southeast. An opposite pattern was observed for bladder cancer mortality risk, which was significantly higher among older mechanics in the Southeast region (MOR: 1.44; 95%CI: 1.02-2.02), but not in the South. Finally, younger and older mechanics in the Southeast region were at higher risk to die by larynx cancer (30-49 years old; MOR:1.87; 95%CI:1.27-2.77 and 50-69 years old; MOR:1.55; 95%CI:1.27-1.90), but such increased risk in the South was observed only among the older ones (MOR: 1,38; 95%CI: 1.20-1.58).

 Mortality odds ratios for selected cancers among mechanics compared with the general population stratified by ethnicity and region of residence are shown in Table 4. Mechanics from South and Southeast regions combined (non-stratified analysis) were at an increased risk to die by oropharynx cancer (white = MOR:2.07, 95% CI:1.79-2.40; non-white = MOR:1.56, 95%CI: 1.29-1.88) and larynx cancer (white = MOR: 1.50, 95%CI: 1.35-1.67; non-white = 1.31, 95%CI: 1.08-1.59), with a slightly higher magnitude among white mechanics. Statistically significant elevated MOR for hypopharynx cancer was only observed among white mechanics (MOR:1.66, 95%CI:1.24-2.22), while only non-white mechanics were at significantly higher risk to die by lung cancer (MOR: 1.17, 95%CI:1.04-1.31), when compared to the general population. Region-stratified analysis showed that in the Southeast region cancer mortality were significantly increased only among white mechanics (Oropharynx = MOR:1.64, 95%CI:1.24-2.16; Larynx = MOR:1.72, 95%CI:1.45-2.04). By contrast, mechanics who lived in the South region were at higher risk to die by the cancers of oropharynx (white = MOR:2.26, 95%CI:1.91-2.68; non-white = MOR:1.52, 95%CI: 1.20-1.93), hypopharynx (white = MOR:1.74, 95%CI:1.20-2.53), larynx (white = MOR:1.38, 95%CI:1.20-1.59; non-white = MOR:1.31, 95%CI: 1.07-1.60), and lung (white = MOR:1.09, 95%CI:1.01-1.18; non-white = MOR:1.23, 95%CI: 1.09-1.39).

Table 5 shows the mortality odds ratios for selected cancers among mechanics compared to the general population, stratified by educational level and region of residence. Combined, mechanics from South and Southeast regions showed significant increased mortality for the cancer of oropharynx, and it was slightly higher among illiterate mechanics (years of schooling: 0 years = MOR: 2.24, 95%CI: 1.01-5.00; 1-7 years = MOR: 1.84, 95%CI: 1.58-2.15; ≥8 = MOR: 2.12, 95%CI: 1.62-2.77), when compared to the general population. In addition, mechanics were also at significantly higher risk to die by hypopharynx cancer, but only among those with ≥8 years of schooling (MOR: 1.95, 95%CI: 1.14-3.33). On the other hand, when compared to the general population, pancreas cancer mortality risk was significantly higher only among mechanics who reported less than 1 year of schooling (MOR: 2.24, 95%CI: 1.21-4.16). Mechanics with 1-7 and ≥8 years of schooling were the ones who showed significantly increased mortality by larynx cancer (1-7 years = MOR: 1.49, 95%CI: 1.29-1.63; ≥8 = MOR: 1.46, 95%CI: 1.18-1.81) and any leukemia (1-7 years = MOR: 1.24, 95%CI: 1.03-1.49; ≥8 = MOR: 1.45; 95%CI: 1.16-1.82). Finally, mechanics with 1-7 years of schooling were also at higher risk to die by lung (MOR: 1.14, 95%CI: 1.07-1.22) and bladder (MOR: 1.28, 95%CI: 1.09-1.51). Region-stratification revealed that only in the Southeast region mechanics with zero years of schooling showed increased mortality by the cancers of oropharynx (MOR: 2.65, 95%CI: 1.19-5.90) and pancreas (MOR: 2.73, 95%CI: 1.37-5.48), when compared with the general population. In addition, only in the Southeast mechanics with 1-7 of schooling were at significantly higher risk to die by lung cancer (MOR: 1.26, 95%CI: 1.16-1.37). On the other hand, mechanics from the South region were the only ones at higher risk to die, at specific education range, by the cancers of larynx (≥8years of schooling = MOR: 1.81, 95%CI: 1.27-2.57), any leukemia (≥8years of schooling = MOR: 2.09, 95%CI: 1.38-3.17), and myeloid leukemia (1-7 years of schooling = MOR: 1.19, 95%CI: 1.10-1.25). Despite all these regional differences in cancer mortality among mechanics according to their educational level, there were also some similarities. For instance, mechanics with both, 1-7 and ≥8years of schooling, were at higher risk to die by oropharynx cancer in the South (1-7 years = MOR: 1.49, 95%CI: 1.09-2.10; ≥8= MOR: 2.31, 95%CI: 1.46-3.67) and Southeast (1-7 years = MOR: 1.92, 95%CI: 1.62-2.28; ≥8= MOR: 2.02, 95%CI: 1.45-2.82) regions, when compared with the general population from same regions and educational level. In addition, mechanics with 1-7 years of schooling showed significant increased mortality, in both regions, for the cancers of larynx (South = MOR: 1.69, 95%CI:1.38-2.07; Southeast = MOR: 1.35, 95%CI:1.17-1.56) and bladder (South = MOR: 1.38, 95%CI: 1.05-1.81; Southeast = MOR: 1.28, 95%CI: 1.04-1.56). 

**Table 1:** Mortality from Selected Cancers Stratified by Sociodemographic Characteristics among Mechanics and the General Population of Brazilian South and Southeast Regions, 2006-2017

	Mechanics	General population	*P*-Value*
	(n = 3095) N (%)	(n = 123,556) N (%)	
Age			
30-49 years	331 (10.7)	12,106 (9.8)	0,83
50-69 years	1,775 (57.4)	64,091 (51.9)	
Education			
None	54 (1.7)	6,988 (5.7)	<0,001
1-7 years	1,811 (58.5)	62,198 (50.3)	
8 + years	706 (22.8)	33,970 (27.5)	
Ethnicity			
White	2,341 (75.6)	93,638 (75.8)	0,49
No-white	691 (22.3)	26,809 (21.7)	
Region			
South	968 (31.3)	46,228 (37.4)	<0,001
Southeast	2,127 (68.7)	77,328 (62.6)	

*, Chi-square’s p-values

**Table 2:** MOR for Selected Cancer among Mechanics and the General Population of Brazilian South and Southeast regions, 2006-2017

		Mechanics	General population	MOR (95%CI)
		(n=3234)	(n= 129913)	
		N (%)	N (%)	
Cancer site				
C10	Oropharynx	274 (8.5)	6,631 (5.1)	1.84 (1.66- 2.11)
C11	Nasopharynx	26 (0.8)	883 (0.7)	1.33 (0.90- 1.97)
C13	Hypopharynx	58 (1.8)	1,882 (1.4)	1.39 (1.07- 1.81)
C25	Pancreas	391(12.1)	17,773 (13.7)	1.00 (0.90- 1.10)
C32	Larynx	456 (14.1)	14,296 (11.0)	1.45 (1.32- 1.59)
C34	Lung	1,399 (43.3)	59,421 (45.7)	1.07 (1.01- 1.12)
C67	Bladder	244 (7.5)	9,526 (7.3)	1.16 (1.02- 1.32)
C91-95	All Leukemias	247 (7.6)	13,144 (10.1)	0.85 (0.75- 0.96)
C92	ML	139 (4.3)	6357 (4.9)	0.99 (0.84- 1.17)

**Table 3:** MOR for Selected Cancers among Mechanics Stratified by Age and Region of Residence, 2006-2017

		30-49 years			50-69 years		
		N_mec_	N_gpop_	MOR (95%IC)	N_mec_	N_gpop_	MOR (95%IC)
Total							
C10	Oropharynx	47	1160	1.66 (1.24-2.22)	195	4418	1.85 (1.60-2.13)
C11	Nasopharynx	7	221	1.29 (0.61-2.75)	16	460	1.45 (0.88-2.39)
C13	Hypopharynx	14	297	1.90 (1.13-3.29)	42	1256	1.40 (1.03-1.90)
C25	Pancreas	47	1824	1.05 (0.79-1.41)	214	9377	0.95 (0.83-1.09)
C32	Larynx	58	1816	1.31 (1.01-1.70)	310	9148	1.42 (1.27 -1.59)
C34	Lung	102	3986	1.05 (0.86-1.27)	792	31975	1.03 (0.97-1.11)
C67	Bladder	9	354	1.04 (0.54-2.01)	98	3326	1.23 (1.00-1.51)
C91-95	All Leukemias	47	2849	0.67 (0.51-0.90)	108	4312	1.04 (0.86-1.26)
C92	ML	26	1382	0.77 (0.52-1.13)	67	2211	1.26 (0.99-1.61)
South region							
C10	Oropharynx	33	829	1.58 (1.11-2.23)	155	3197	1.91 (1.67-2.19)
C11	Nasopharynx	6	148	1.60 (0.71-3.62)	12	318	1.48 (0.83-2.63)
C13	Hypopharynx	10	185	2.14 (1.13-4.03)	28	801	1.37 (0.94-2.00)
C25	Pancreas	29	1203	0.95 (0.66-1.37)	150	6061	0.97 (0.83-1.14)
C32	Larynx	32	1196	1.06 (0.74-1.50)	216	6177	1,38 (1.20-1.58)
C34	Lung	64	2454	1.03 (0.80-1.32)	520	18869	1.08 (0.99-1.18)
C67	Bladder	6	224	1.06 (0.47-2.38)	64	2173	1.15 (0.90-1.48)
C91-95	All Leukemias	36	2076	0.68 (0.49-0.95)	76	2903	1.03 (0.82-1.29)
C92	ML	21	992	0.84 (0.54-1.29)	53	1525	1.36 (1.04-1.79)
Southeast region							
C10	Oropharynx	14	331	1.88 (1.10-3.21)	40	1221	1.60 (1.17-2.19)
C11	Nasopharynx	1	73	0.61 (0.08-4.38)	4	142	1.37 (0.51-3.70)
C13	Hypopharynx	4	112	1.59 (0.59-4.30)	14	455	1.50 (0.88-2.55)
C25	Pancreas	18	621	1.30 (0.76-2.06)	64	3316	0.94 (0.73-1.20)
C32	Larynx	26	620	1.87 (1.27-2.77)	94	2971	1.55 (1.27-1.90)
C34	Lung	38	1532	1.10 (0.80-1.52)	272	13106	1.01 (0.90-1.14)
C67	Bladder	3	130	1.02 (0.33-3.22)	34	1153	1.44 (1.02-2.02)
C91-95	All Leukemias	11	773	0.63 (0.35-1.14)	32	1409	1.01 (0.78-1.57)
C92	ML	5	390	0.57 (0.24-1.37)	14	686	0.99 (0.59-1.69)

N_mec_, mechanic population; N_gpop_, general population

**Table 4:** MOR for Selected Cancers among Mechanics Stratified by Race and Brazilian Southeast and South Region, 2006-2017

		White			No-white		
		N_mec_	N_gpop_	MOR (95%IC)	N_mec_	N_gpop_	MOR (95%IC)
ICD							
Total							
C10	Oropharynx	194	4235	2.07 (1.79-2.40)	77	2251	1.56 (1.29 - 1.88)
C11	Nasopharynx	18	620	1.30 (0.82-2.08)	8	241	1.51 (0.75 - 3.05)
C13	Hypopharynx	47	1,275	1.66 (1.24-2.22)	11	559	0.89 (0.49 - 1.62)
C25	Pancreas	304	13,566	1.01 (0.90-1.13)	75	3,727	0.91 (0.73 - 1.15)
C32	Larynx	338	10,163	1,50 (1.35-1.67)	109	3,785	1.31 (1.08 - 1.59)
C34	Lung	1,070	46,198	1.04 (0.98-1.11)	301	11,743	1.17 (1.04 - 1.31)
C67	Bladder	181	7,732	1.05 (0.91-1.22)	56	15,68	1.63 (1.25 - 2.12)
C91-95	All Leukemias	189	9,849	0.86 (0.75-0.99)	54	2,935	0.84 (0.64 - 1.09)
C92	ML	102	4,774	0.96 (0.79-1.17)	35	1,402	1.14 (0.81 - 1.59)
South region							
C10	Oropharynx	143	2,614	2.26 (1.91-2.68)	70	1,995	1.52 (1.20 - 1.93)
C11	Nasopharynx	13	377	1.42 (0.75 - 2.46)	8	212	1.63 (0.81 - 3.31)
C13	Hypopharynx	29	686	1.74 (1.20 - 2.53)	11	461	1.03 (0.57 - 1.88)
C25	Pancreas	189	7,991	0.97 (0.84 - 1.12)	61	3,123	0.84 (0.66 - 1.09)
C32	Larynx	202	6,043	1.38 (1.20 - 1.59)	99	3,285	1.31 (1.07 - 1.60)
C34	Lung	647	24,496	1.09 (1.01 - 1.18)	270	9,538	1.23 (1.09 - 1.39)
C67	Bladder	118	4,674	1.04 (0.87 - 1.25)	49	1,317	1.61 (1.21 - 2.14)
C91-95	All Leukemias	127	8,542	0.61 (0.51 - 0.73)	47	5,756	0.35 (0.27 - 0.47)
C92	ML	78	3,059	1.05 (0.84 - 1.31)	31	1,224	1.10 (0.77 - 1.57)
Southeast region							
C10	Oropharynx	51	1,621	1.64 (1.24 - 2.16)	7	256	1.79 (0.84 - 3.78)
C11	Nasopharynx	5	243	1.07 (0.44 - 2.59)	0	29	-
C13	Hypopharynx	18	589	1.59 (0.99 - 2.53)	0	98	-
C25	Pancreas	115	5,575	1.07 (0.89 - 1.29)	14	604	1.52 (0.90 - 2.57)
C32	Larynx	136	4,120	1.72 (1.45 - 2.04)	10	500	1.31 (0.70 - 2.44)
C34	Lung	423	21,702	1.01 (0.92 - 1.11)	31	2,205	0.92 (0.64 - 1.30)
C67	Bladder	63	3,058	1.07 (0.83 - 1.37)	7	251	1.82 (0.86 - 3.86)
C91-95	All Leukemias	62	4,093	0.78 (0.61 - 1.01)	7	435	1.05 (0.50 - 2.21)
C92	ML	24	1,715	0.73 (0.49 - 1.08)	4	178	1.47 (0.55 - 3.94)

N_mec_, mechanic population; N_gpop_, general population

**Table 5:** Mortality Odds Ratio (MOR) for Selected Cancers among Mechanics Stratified by Schooling and Brazilian Southeast and South Region, 2006-2017

	0 years			1-7 years			≥ 8 years		
	N_mec_	N_gpop_	MOR (95%IC)	N_mec_	N_gpop_	MOR (95%IC)	N_mec_	N_gpop_	MOR (95%IC)
ICD									
Total									
C10 Oropharynx	6	480	2.24 (1.01 - 5.00)	170	3,790	1.84 (1.58 - 2.15)	55	1,210	2.12 (1.62 - 2.77)
C11 Nasopharynx	0	48	-	13	423	1.26 (0.73 - 2.19)	6	281	0.99 (0.44 - 2.23)
C13 Hypopharynx	0	112	-	32	1,105	1.19 (0.84 - 1.69)	14	334	1.95 (1.14 - 3.33)
C25 Pancreas	10	802	2.24 (1.21 - 4.16)	205	8,017	1.05 (0.91 - 1.20)	102	6,121	0.77 (0.64 - 0.94)
C32 Larynx	6	948	1.13 (0.51 - 2.52)	282	8,015	1.49 (1.29 - 1.63)	84	2,680	1.46 (1.18 - 1.81)
C34 Lung	23	3,536	1.17 (0.78 - 1.75)	839	30,296	1.14 (1.07 - 1.22)	310	15,933	0.90 (0.81 - 1.01)
C67 Bladder	4	559	1.28 (0.48 - 3.41)	150	4,792	1.28 (1.09 - 1.51)	59	2,736	1.02 (0.77 - 1.30)
C91-95 All Leukemias	5	848	1.05 (0.44 - 2.54)	120	3,964	1.24 (1.03 - 1.49)	76	2,427	1.45 (1.16 - 1.82)
C92 ML	2	188	1.90 (0.47 - 7.64)	61	2,606	0.96 (0.74 - 1.24)	51	2,469	0.96 (0.73 - 1.27)
South region									
C10 Oropharynx	0	128	-	34	1,178	1.49 (1.06 - 2.10)	19	376	2.31 (1.46 - 3.67)
C11 Nasopharynx	0	16	-	2	149	0.69 (0.17 - 2.79)	2	72	1.27 (0.31 - 5.17)
C13 Hypopharynx	0	45	-	10	463	1.11 (0.60 - 2.08)	5	115	1.99 (0.81 - 4.86)
C25 Pancreas	2	347	1.39 (0.35 - 5.56)	80	3,485	1.19 (0.95 - 1.48)	33	1,656	0.91 (0.65 - 1.28)
C32 Larynx	2	320	1.51 (0.38 - 6.04)	95	2,918	1.69 (1.38 - 2.07)	32	811	1.81 (1.27 - 2.57)
C34 Lung	5	1,692	0.71 (0.30 - 1.68)	297	14,384	1.07 (0.95 - 1.20)	101	4,716	0.98 (0.80 - 1.19)
C67 Bladder	0	233	-	54	2,022	1.38 (1.05 - 1.81)	14	740	0.86 (0.51 - 1.46)
C91-95 All Leukemias	2	313	1.55 (0.39 - 6.18)	36	1,341	1.38 (0.99 - 1.92)	23	502	2.09 (1.38 - 3.17)
C92 ML	1	65	3.72 (0.52 - 26.73)	13	926	1.19 (1.10 - 1.25)	13	654	0.91 (0.52 - 1.57)
Southeast region									
C10 Oropharynx	6	352	2.65 (1.19 - 5.90)	136	2,612	1.92 (1.62 - 2.28)	36	834	2.02 (1.45 - 2.82)
C11 Nasopharynx	0	32	-	11	274	1.48 (0.81 - 2.70)	4	209	0.89 (0.33 - 2.41)
C13 Hypopharynx	0	67	-	22	642	1.26 (0.82 - 1.93)	9	219	1.92 (0.99 - 3.75)
C25 Pancreas	8	455	2.73 (1.37 - 5.48)	125	4,532	1.01 (0.85 - 1.21)	69	4,465	0.72 (0.57 - 0.91)
C32 Larynx	4	628	0.98 (0.37 - 2.62)	187	5,097	1.35 (1.17 - 1.56)	52	1,869	1.30 (0.99 - 1.72)
C34 Lung	18	1,844	1.52 (0.96 - 2.40)	542	15,912	1.26 (1.16 - 1.37)	209	11,217	0.87 (0.76 - 0.99)
C67 Bladder	4	326	1.90 (0.71 - 5.07)	96	2,770	1.28 (1.04 - 1.56)	45	1,996	1.05 (0.79 - 1.42)
C91-95 All Leukemias	3	535	0.87 (0.28 - 2.69)	84	2,623	1.18 (0.95 - 1.46)	53	1,925	1.28 (0.98 - 1.69)
C92 ML	1	123	1.25 (0.18 - 8.96)	48	1,680	1.05 (0.79 - 1.40)	38	1,815	0.98 (0.71 - 1.35)

N_mec_, mechanic population; N_gpop_, general population

## Discussion

Our findings show that Brazilian mechanics may be at higher risk to die by selected malignant neoplasms. Accordingly, Vaughan (1989) also observed an increased risk of oropharynx and hypopharynx cancers associated to industrial mechanic occupation (OR = 31.0; IC95%: 3.0-315.1), but not to vehicle mechanics (OR = 2.50; IC95%: 0.80-8.50). On the other hand, in another study, vehicle mechanics were at higher risk to die by the cancers of oropharynx and oral cavity combined (OR = 2.45; IC95%: 1.14-5.27), especially those who reported ten years or more in the job (OR = 7.38; IC95%: 1.88-28.98) (Andreotti et al., 2006). In addition, Pukkala et al., (2009), have reported a slightly increased risk of larynx cancer (OR= 1.12; IC95%:1.06-1.18), but not to pharynx, oropharynx and nasopharynx cancers (OR = 1.00; IC95%: 0.93-1.07; OR = 1.02, IC95%: 0.92-1.14; OR = 0.97, IC95%: 1.02-1.15), among male mechanics. A meta-analysis of studies on occupational cancer revealed that mechanics in general (SRR= 1.21, IC95%: 1.12-1.31), and more specifically motor mechanics (SRR= 1.27, IC95%: 1.10-1.46) showed a modest increased risk of bladder cancer (Reulen et al., 2008). Alguacil et al., (2000) found elevated, although non-significant, pancreatic cancer risk among machinery mechanics with more than 20 years of working (OR = 3.4, IC95%: 0.6-18.2). Asbestos is strongly associated to lung cancer. Motor vehicle mechanics are potentially exposed to asbestos during asbestos-containing brakes repair. Although in our study we could not differentiate vehicle mechanics from others, we observed a slightly increased mortality from lung cancer in non-white mechanics in the South region. However, the mechanic occupation has been inconsistently associated with lung cancer, as some ’80s and ‘90s studies have shown positive associations (Zahm et al., 1989; Matos et al., 1998; Milne et al., 1983; Finkelstein, 1989), while recent studies have not confirmed it (Goodman et al., 2004; Garshick et al., 2008; Strand et al., 2015; Laden et al., 2004). Benzene is one of the most well stablished environmental risk factor for acute myeloid leukemia (IARC, 2018). Exposure of motor vehicle mechanics to benzene can occur during gasoline-related tasks (Willians and Mani, 2015). In the current study, we observed elevated mortality from myeloid leukemia among 50-69 years old mechanics who lived in the South, but not in the Southeast region. In fact, three previous studies have also shown a significant association between increased leukemia mortality and vehicle mechanics (Schwartz, 1987; Hunting et al., 1995; Ji et al., 2005), an another one between acute myeloid leukemia and automotive service technicians and mechanics, but without statistics significance (Tsai et al., 2014). In the opposite direction, Silva et al., (2000) did not find any increase in leukemia mortality among military motor and aviation mechanics, craftsmen mechanics, and metallurgists.

The lack of knowledge on potential risks associated to the chemicals used on a daily basis by mechanics can intensify their exposure to these hazardous substances. For instance, in Brazilian smaller garages over half of motor vehicle mechanics don’t use personal protective equipments (Binder et al., 2001; Novais, 2015). In addition, poor ventilation is a typical condition in Brazilian vehicle repair shops or garages (Binder et al., 2001; Santos et al., 2010). In our study, mortality risk by bladder, lung, and pancreas cancers was higher among less educated mechanics than highly educated ones, when compared to the general population. 

Studies based on mortality records from the Brazilian Mortality Database involves some recognisable limitations. For instance, the lack of information on well known risk factors for the cancers included in the study, did not allow us to test them for potential confounding in our analyses. Alcohol use is an independent risk factor for pharyngeal and laryngeal cancer (Dal Maso et al., 2002; Znaor et al., 2003; Ansay-Moghaddam et al., 2009; Okekpa et al., 2019). On the other hand, smoking is the major risk factor for lung cancer, but it is suspected to also play a relevant role in acute myeloid leukemia, as well as in the development of upper respiratory tract cancers (Hecht, 1999; Vineis et al., 2004; Furrukh, 2013). Infections by human papillomavirus (HPV) and Epstein-Barr virus (EBV) are strongly associated with oropharynx, nasopharynx and larynx cancers (D’Souza and Dempsey, 2011; Young and Dawson, 2014; Gupta and Johnson, 2014). Consumption of red meat and hot food and beverages has also been positively associated with the development of pharynx and larynx cancers (De Stefani et al., 1999, Chuang et al., 2012). Despite the lack of information about these known risk factors on the Brazilian mortality information system, there is no evidence that mechanics and the general population are biased exposed to them in a way that could distort the associations observed in our study. In our study, missing data on education (years of schooling) were 17% for both mechanics and the general population. Since these comparison groups differed in terms of education (Table 1), it is possible that these missing data also have followed this unbalanced distribution and could distort the true association between mechanic occupation and mortality risks for the studied cancers.

Despite the above limitations, our study used a large dataset of death certificates that allowed us to perform robust analyses. In addition, Brazilian Mortality Database completeness and accuracy have been attested in previous studies, especially for the South and Southeast regions (Lima and Queiroz, 2014). Finally, the literature on cancer among mechanics are scarce and old.

In conclusion, the results of this study suggest that Brazilian mechanics may be at higher risk to die by myeloid leukaemia, bladder, lung, pancreas, and upper respiratory tract cancers. However, the controversy about the risk of cancer among mechanics remains and supports the need to explore this hypothesis through further analytical studies.
